# A new genus of Strepsiptera, *Rozenia* gen. n. (Stylopidae), a parasite of bee genera *Acamptopoeum* and *Calliopsis* (Andrenidae, Panurginae, Calliopsini)

**DOI:** 10.3897/zookeys.442.7747

**Published:** 2014-09-23

**Authors:** Jakub Straka, Katerina Jůzová, Jan Batelka

**Affiliations:** 1 Department of Zoology, Charles University in Prague, Viničná 7, CZ-12844 Praha 2, Czech Republic; 2 Nad vodovodem 16, CZ-100 00 Praha 10, Czech Republic

**Keywords:** New genus, new species, Apoidea, host-parasite association, morphology, chaetotaxy, description, South America

## Abstract

A new Strepsiptera genus from South America is described, *Rozenia* **gen. n.**, with three new species: *Rozenia calliopsidis* **sp. n.** (type species), *Rozenia peruana* **sp. n.** and *Rozenia platicephala* **sp. n.** These three new species are parasites of bees belonging to the tribe Calliopsini (Andrenidae, Panurginae). *Rozenia calliopsidis* **sp. n.** is a parasite of the bee genus *Calliopsis* Smith, 1853 and *Rozenia peruana* **sp. n.** and *Rozenia platicephala* **sp. n.** are parasites of the bee genus *Acamptopoeum* Cockerell, 1905. Diagnoses and descriptions of female puparia are presented for all three species. Diagnoses and descriptions of first instars (triungulinids) are presented for *Rozenia calliopsidis* **sp. n.** and *Rozenia platicephala* **sp. n.** The first case of increased number of setae on the body of the first instars and augmentation of chaetotaxy of Strepsiptera are discussed.

## Introduction

Stylopization of a bee from the tribe Calliopsini (Andrenidae, Panurginae) was recorded for the first time as early as 1931 (Schwarz 1931). Another finding was presented shortly afterwards from Argentina (Hofeneder and Fulmek 1943). Both records were repeated in the literature several times under various combinations of the host names (Hofeneder and Fulmek 1943, Hofeneder 1949, 1952) that recently belong to the genus *Acamptopoeum* Cockerell, 1905. However, no other record of stylopization of a bee from the tribe Calliopsini has been published since that time.

Members of the tribe Calliopsini are not the only known stylopized panurgine bees. Pierce (1904) published a note on a stylopized bee from the tribe Protandrenini and subsequently added data on stylopization of a wider range of species from the tribe Protandrenini from North America (Pierce 1909). All these North American panurgine hosts of Strepsiptera belong to the genus *Pseudopanurgus* Cockerell, 1897; Strepsiptera parasitizing Protandrenini are also known from South America (Holmberg 1921, Ogloblin 1947, Kogan 1989). These hosts belong to the genera *Anthrenoides* Ducke, 1907, *Psaenythia* Gerstaecker, 1868 and *Rhophitulus* Ducke, 1907. There are also two other genera of Panurgini known to be hosts of Strepsiptera in the Palearctic region. The first note about stylopized *Panurgus* Panzer, 1806 (Panurgini) was made by Morice (1913) and later, Ogloblin (1925) recorded Strepsiptera from the genus *Panurginus* Nylander, 1848.

All described Strepsiptera, which parasitize panurgine bees, were placed in the genus *Crawfordia* Pierce, 1908. All bees were from Neotropical, Nearctic, or Palearctic regions. Regarding the Strepsiptera that parasitize bees from the tribe Calliopsini, no taxon has ever been described, even though the host-parasite association has been known for more than eighty years. Here we present a new genus of Strepsiptera associated with the bee tribe Calliopsini, with a description of three new species. We compare the morphology of female puparia and the first instars with other genera of Strepsiptera, and particularly with species parasitizing bees (Stylopidae), especially other members of the bee subfamily Panurginae.

## Methods

### Collections

Material from the following public and private collections was examined:

AMNH American Museum of Natural History, Jerome G. Rozen Jr., (New York, USA);

JSPC Jakub Straka personal collection, (Praha, Czech Republic);

KUNHM Natural History Museum, Division of Entomology, University of Kansas, Michael S. Engel, (Lawrence, Kansas, USA).

### Preparation of material

All host individuals were first relaxed and then dissected. Females and first instar larvae were removed from the host body. Strepsiptera females studied for morphology were cleared using proteinase: a mixture of lysis buffer and proteinase K (Quiagen) was heated to 56 °C. The lysis procedure took several hours or overnight. Cleared specimens were cleaned in water several times and then stored in vials with glycerol. A drawing tube (camera lucida) was attached to an Olympus BX40 light microscope and an Olympus SZX9 binocular microscope and used for morphological studies and drawings. Temporary slides were prepared with glycerol.

First instar larvae were removed from the female’s body. Specimens used for morphological studies were prepared using the same method as females, except for scanning electron microscopy (SEM). For SEM, first instars were stored in 96% ethanol and subsequently dehydrated in 100% ethanol for 5–10 minutes and then acetone for 5 minutes. Dehydrated specimens were critical-point dried and coated with gold. For scanning electron microscopy we used a JEOL 6380 LV.

### Morphology and terminology

External structures of first instars and female puparia are described. The mature or teneral female is presented inside the external puparium, but these have never been used for species descriptions. The body is weakly sclerotized and, in addition to the number of birth organs (tubae proliferae), lacks any practical characters.

Morphological terminology of female puparium follows Kinzelbach (1971) except:

basal band pigmented external part of abdominal segment I, usually distinct on ventral side;

cephalic ridge intersegmental ridge between head and prothorax on ventral side;

cintum constriction dividing inner and outer part of tergum I;

head corner lateral extensions of head behind brood opening on ventral side;

oral ridge mouth sensu Kinzelbach (1971);

prothoracic ridge intersegmental ridge between prothorax and mesothorax on ventral side.

Terminology of first instar larvae follows Pohl (2000, 2002) except:

interstitial row of setae additional row of setae between submedian and supralateral row on thoracic tergites.

Specimens of strepsipterans are indicated by the following abbreviations: EMP – empty male puparium; MP – male puparium; FP – female puparium; L1 – first instar larva.

### Description style

All newly described species were labeled as follows: “HOLOTYPUS FP, name of taxon sp. nov., Jakub Straka det. 2014” on red card; paratypes analogously on yellow card. Precise label data on locality are cited for the holotypes. Separate lines on a label are indicated by a slash “/” and separate card labels are indicated by a double slash “//”.

Information on the distribution and etymology of names are provided in separate paragraphs for each species. An overview of the host-parasite associations with published and updated host names is presented in Table 1. Information concerning host stylopization without classification of the Strepsiptera and other information are within the notes.

**Table 1.** Summary of host associations for *Rozenia* gen. n. All hosts belong to bees (Apoidea) of the family Andrenidae; (as) host published under the different combination or misidentification; (*) host association corroborated in this study. Valid names are in bold.

Parasite	Host
**Strepsiptera**	**Hymenoptera**
**Stylopidae**	**Panurginae** Leach, 1805 (Apoidea: Andrenidae Latreille, 1802)
***Rozenia*** gen. n.	**Calliopsini** Robertson, 1922
***Rozenia calliopsidis*** sp. n.	*Calliopsis (Liopoeum) mendocina (Jörgensen, 1912)
	*Calliopsis (Liopoeum) trifasciata (Spinola, 1851)
***Rozenia peruana*** sp. n.	*Acamptopoeum vagans (Cockerell, 1926)
***Rozenia platicephala*** sp. n.	*Acamptopoeum submetallicum (Spinola, 1851)
	as *Liopoeum submetallicum* (Spinola, 1851) (Schwarz 1931, Hofeneder and Fulmek 1943)
***Rozenia*** sp.	***Acamptopoeum argentinum*** (Friese, 1906)
	as *Perdita argentina* Friese, 1906 (Hofeneder and Fulmek 1943)
	as Calliopsis (Parafriesea) argentina (Friese, 1906) (Hofeneder 1952)

## Genus and species descriptions

### Rozenia gen. n.

Taxon classificationAnimaliaStrepsipteraStylopidae

http://zoobank.org/00957F90-4A0F-4ACB-AAB5-9CFF8B9A303A

#### Type species.

*Rozenia calliopsidis* sp. n.

#### Diagnosis.

**Female.** *Rozenia* gen. n. differs from other genera of the family Stylopidae in having only four abdominal segments. Similarly to the genus *Crawfordia* Pierce, 1908, canalis prolifer of *Rozenia* gen. n. is with a single median tuba prolifera present on segments II-IV of the abdominal part of female. However, tuba prolifera III of *Rozenia* gen. n. is positioned on the posterior half of abdominal segment IV, but in the middle of segment IV in *Crawfordia*, which possesses also rudimentary segment V. Abdomen of other genera of the family Stylopidae is composed by higher number of segments.

**Female puparium.** Brood opening of the new genus is very wide, almost from side to side, about four times wider than intermandibular distance, or more in *Rozenia* gen. n. Brood opening is usually much narrower in other genera of the family Stylopidae. Narrow head corners are produced laterally beyond prothorax; this feature causes head to be wider than distal part of prothorax and side of cephalothorax is not continuously diverging posteriorly. This character is developed in *Eurystylops* Bohart, 1943 and some species of the genus *Crawfordia*. Head corners are relatively long, but not as long as in *Crawfordia*, which possess head corners longer than half of cephalothorax. In *Rozenia* gen. n. head corners are as long as head dorsally, but *Crawfordia* has much longer head corners than head dorsally. Mandibles extending from the head contour in ventral view. In contrast to *Crawfordia*, intersegmental ridges are not developed in *Rozenia* gen. n.

**First instar.** First instars of *Rozenia* gen. n. differ substantially from other genera by having setae of submedian row on thorax as well as on abdominal segments. Caudal setae are distinctly longer than body. Both these characters are unique among all Strepsiptera. *Rozenia* gen. n. does not have spinulae on posterior margin of thoracic tergites as in Xenidae, Halictophagidae, or Elenchidae. These spinulae are developed on posterior margin of thoracic tergites in all other genera of the family Stylopidae. Ventral sublateral bristle is missing on sternum IX in *Rozenia* gen. n., but probably present in all other genera of Strepsiptera. Posterior margin of labiomaxilary area continuous in *Rozenia* gen. n., but emarginated in *Crawfordia*, *Halictoxenos* and *Stylops* (and probably also in other Stylopidae).

#### Description.

**Female.** Canalis prolifer on abdominal segments I-IV, segment V absent. Single median tuba prolifera on segments II-IV, tuba prolifera on segment IV positioned in posterior half of segment.

**Female puparium.** Head corners (on ventral side) extending posteriorly as far as head posterior margin on dorsal side; head corners distinct, narrow, forming a lamella on frontal part of cephalothorax, produced laterally beyond prothorax, this feature causes head to be wider than distal part of prothorax and side of cephalothorax is not continuously diverging posteriorly; head corners elevated ventrally over intermandibular part of head, but not over prothorax; brood opening wide, distinctly wider than distance between mandibles; mandibles variable in size, but at least the tip is extending from the head contour in ventral view. Intersegmental ridges not developed; anterior margin of mesothorax ill-defined, but transverse and does not extend forward; spiracles positioned distally above prominent spiracular corners, close to middle of cephalothorax. Prothorax ventrally pigmented, not lighter than head corners.

**Male.** Unknown.

**First instar.** Body rounded; thorax approx. half of entire body length (caudal setae not included); caudal setae distinctly longer than body length. Head strongly reduced ventrally; maxilla with single seta; mandibles and labrum overlapping outline of body; labium fused to maxillae forming labiomaxillary area, its posterior margin continuous, not emarginated.

Each segment of thorax bears at least two pairs of setae dorsally and laterally close to posterior margin, forming submedian and lateral rows of setae. Posterior margins of thoracic tergites smooth. Coxae broad, ovate; three coxal teeth at anterior part of each coxa, all divided into two to four tips; one coxal bristle divided at least into two tips; up to five cuticular outgrowths laterally from coxal teeth and one very short seta anteriorly from cuticular outgrowths; one very short seta at posterior part of coxa. Each trochanterofemur with femoral spur bifid at tip; up to six cuticular outgrowths and one short seta anteriorly and posteriorly on femur; each tibia with five tibial spurs and small projections at distal end of tibia. Tarsi of fore and mid legs enlarged and elongated; tarsi of hind legs rod-like and elongated. Sternal figs broad and smooth on surface (paired setae missing).

Abdomen with rows of setae similar to those present on thorax. Abdominal segment X extremely shortened and fused to segment IX; segment XI split into two parts and restricted to ventral base of caudal setae; segment XI with one particularly long caudal seta and short lateral caudal seta. Posterior margins of abdominal tergites smooth except laterally, spinulae not immersed; posterior margin of abdominal sternites with spinulae, spinulae not immersed; segment IX with only two spinulae, ventral sublateral bristle is missing.

#### Hosts.

Bees of the genera *Acamptopoeum* and *Calliopsis*.

#### Etymology.

Named in honor of the excellent bee expert, teacher of generations of bee students and a friendly and knowledgeable man, Jerome G. Rozen Jr. (American Museum of Natural History, New York, USA). J.G. Rozen Jr., collected most of the specimens of all three new species used for the descriptions.

### Rozenia calliopsidis sp. n.

Taxon classificationAnimaliaStrepsipteraStylopidae

http://zoobank.org/DABEEEE4-1BEF-4764-812D-054FDA6D8FD3

Figures 1 , 3 , 6 , 9 , 12 , 14 , 17 , 19 , 20 , 22

#### Material examined.

Holotype female puparium, in a separate microvial on the same pin as host. Original label: “CHILE: R.M.: Chacabuco / Caleu, nr. Cerro del Robie / 33°00'49"S, 70°58'59"W / 30 Nov 2004, J. S. Ascher, / A. Y. Kawahara, C. Espina”. 1 FP, host: Calliopsis (Liopoeum) trifasciata (Spinola, 1851), ♂, AMNH coll. (code: AMNH_BEE 00036534).

Paratypes: ARGENTINA: Salta prov.: Cafayete, 14.xi.1993, 1 FP, host: Calliopsis (Liopoeum) mendocina (Jörgensen, 1912) ♀, JG and BL Rozen leg., AMNH coll. (AMNH_BEE 00036520), ditto, 1 FP (AMNH_BEE 00036521), ditto (AMNH_BEE 00036522); Catamarca prov.: El Desmonte, 7.xi.1989, 1 FP, host: *Calliopsis mendocina* ♂, JG Rozen and A Roig-Alsina leg., AMNH coll. (AMNH_BEE 00036523), ditto 2 FPP (AMNH_BEE 00036524), San Fernando, 3.–6.xi.1989, 1 FP, host: *Calliopsis mendocina* ♀, JG Rozen and A Roig-Alsina leg., AMNH coll. (AMNH_BEE 00036525), ditto, 5.xi.1991, 1 FP, host: *Calliopsis mendocina* ♂, JG Rozen, LE Peña and A Ugarte leg., AMNH coll. (AMNH_BEE 00036529), ditto, 15.xi.1993, 1 FP, host: *Calliopsis mendocina* ♂, JG and BL Rozen leg., AMNH coll. (AMNH_BEE 00036528), Tinogasta 35 km SE, 28.xi.1989, 1 FP, host: *Calliopsis mendocina* ♂, JG Rozen and A Roig-Alsina leg., AMNH coll. (AMNH_BEE 00036526), Copacabana, 30.xi.1993, 1 FP, host: *Calliopsis mendocina* ♂, JG Rozen leg., AMNH coll. (AMNH_BEE 00036527), Punta de Balasto 3–15 km WSW, 25.xi.1993, 1 FP, host: *Calliopsis mendocina* ♀, JG Rozen leg., AMNH coll. (AMNH_BEE 00036530); Tucumán prov.: Amaichá del Valle, 6.iii.1990, 1 MP with pupa, host: *Calliopsis mendocina* ♂, JG Rozen leg., AMNH coll. (AMNH_BEE 00036532); Rio Negro prov.: El Bolson, 17.ii.1960, 1 FP, >50 L1, host: *Calliopsis trifasciata* ♂, A Kovacs leg., AMNH coll. (AMNH_BEE 00036533); Neuquén prov.: Junín de los Andes, 21.–23.ii.2004, 2 FPP, host: *Calliopsis trifasciata* ♀, J Straka leg. and det., JSPC coll.; CHILE: Apoquindo, Santiago, 1FP, host: *Calliopsis trifasciata* ♂, date and collector not indicated, KUNHM coll. (SEMC1008235); Macul, SE Santiago, 5.xi.1974, 2 FPP, host: *Calliopsis trifasciata* ♀, LE Peña leg., AMNH coll. (AMNH_BEE 00036536); Petorca prov.: Las Palmas tunnel, 18.x.1994, 2 FPP, host: *Calliopsis trifasciata* ♂, JG Rozen, Quinter and JS Ascher leg., AMNH coll. (AMNH_BEE 00036535). Other material examined: Salta prov.: El Carmen, 27 km S Molinos, 1900 m, 6.x.1968, 1 EMP, host: *Calliopsis mendocina* ♀, LE Peña leg., AMNH coll. (AMNH_BEE 00036519). If not indicated otherwise, bee hosts identified by JS Ascher.

#### Diagnosis.

**Female puparium.** *Rozenia calliopsidis* sp. n. differs from other species of the genus by a narrower head with large mandibles. Brood opening turned backwards laterally, very close to posterior margin of mandible and continued as cephalic ridge. In other species, the brood opening fluently transforms into cephalic ridge and forms an arcuate line. Spiracular corners of this species are weakly prominent, obtuse, not triangular. Whole cephalothorax is darker than in *Rozenia platicephala* sp. n. and *Rozenia peruana* sp. n.

**Figures 1–2.**
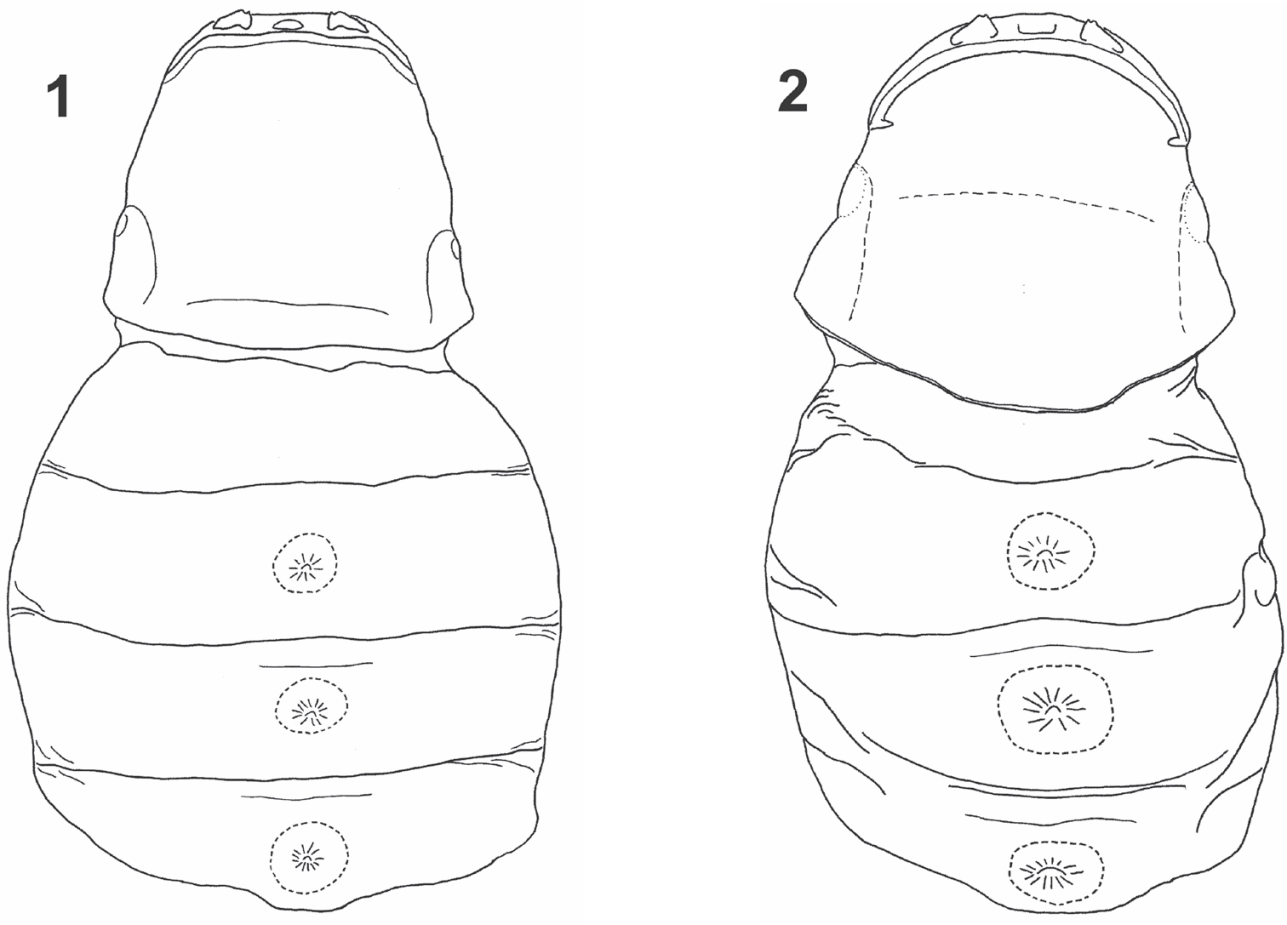
Female puparium, cephalothorax, with canalis prolifer of female, ventral view. **1** *Rozenia calliopsidis* sp. n. **2** *Rozenia platicephala* sp. n.

**Figures 3–11.**
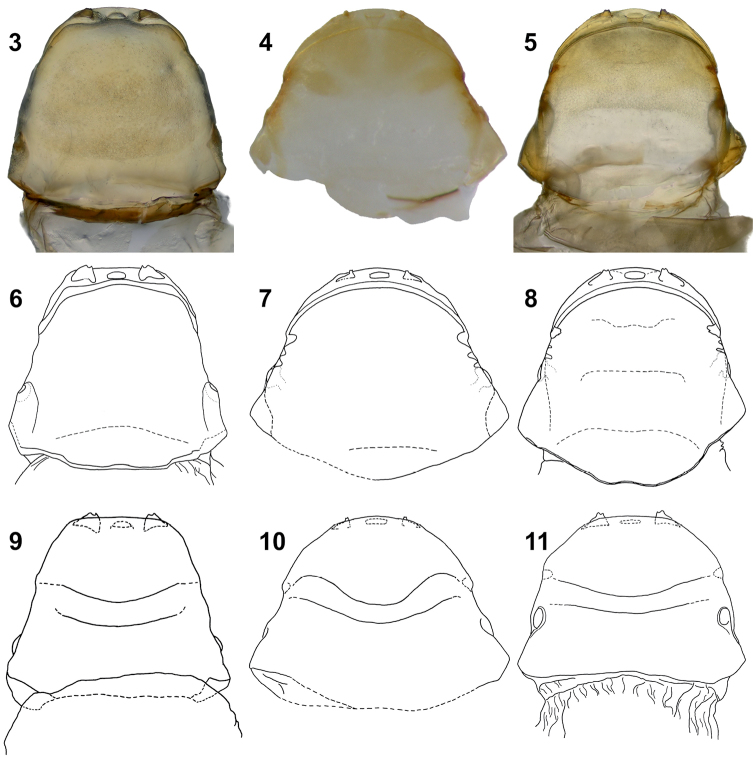
Female puparium, cephalothorax. **3, 6, 9** *Rozenia calliopsidis* sp. n., ventral and dorsal view **4, 7, 10** *Rozenia peruana* sp. n., ventral and dorsal view **5, 8, 11** *Rozenia platicephala* sp. n., ventral and dorsal view.

**First instar.** Shape of body narrower than in *Rozenia platicephala* sp. n. Ratio of body length and width is on average 2.3. Ratio of body length and length of caudal setae is 0.74–0.96. Caudal setae are shorter than in *Rozenia platicephala* sp. n.

Head dorsally with seven pairs of setae compared to six and usually shorter in *Rozenia platicephala* sp. n. Labrum is not emarginated in the middle in contrary to *Rozenia platicephala* sp. n. Labiomaxillary area more rounded than in *Rozenia platicephala* sp. n., acute posteriorly.

Each segment of thorax bears only two pairs of setae dorsally and laterally, forming submedian and lateral row of setae, both rows continue on abdomen, interstitial and supralateral rows of setae missing. Posterior margin of abdominal tergites with more spinulae laterally than in *Rozenia platicephala* sp. n. These spinulae are visible in dorsal view.

Sternal figs are broad and smooth on surface, posterior margin with fringe of long spinulae in contrast to smooth margins of *Rozenia platicephala* sp. n. Precoxal pleural membrane of prothorax covered with transverse row of microtrichiae and precoxal pleural membrane of meso and metathorax with two cuticular processes laterally and medially.

Coxal teeth are usually divided into three to five tips; coxal bristle is divided into four or five tips on foreleg and into two tips on middle and hind legs; this bifurcation is more extensive in comparison to *Rozenia platicephala* sp. n. Coxa and trochanterofemur with more cuticular outgrowths in comparison to *Rozenia platicephala* sp. n.

**Figures 12–15.**
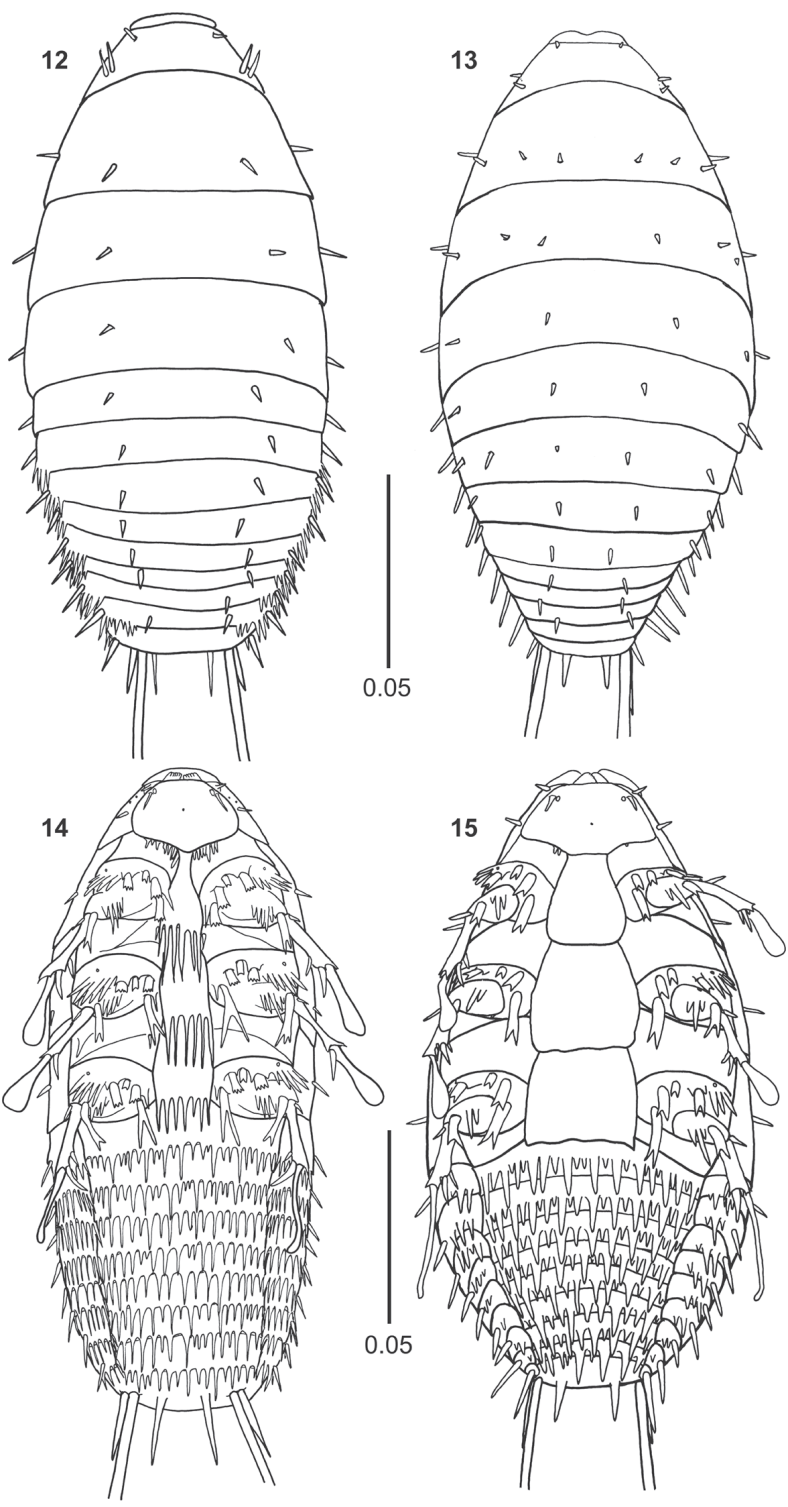
First instars, dorsal and ventral view. **12, 14** *Rozenia calliopsidis* sp. n. **13, 15** *Rozenia platicephala* sp. n.

**Figures 16–19.**
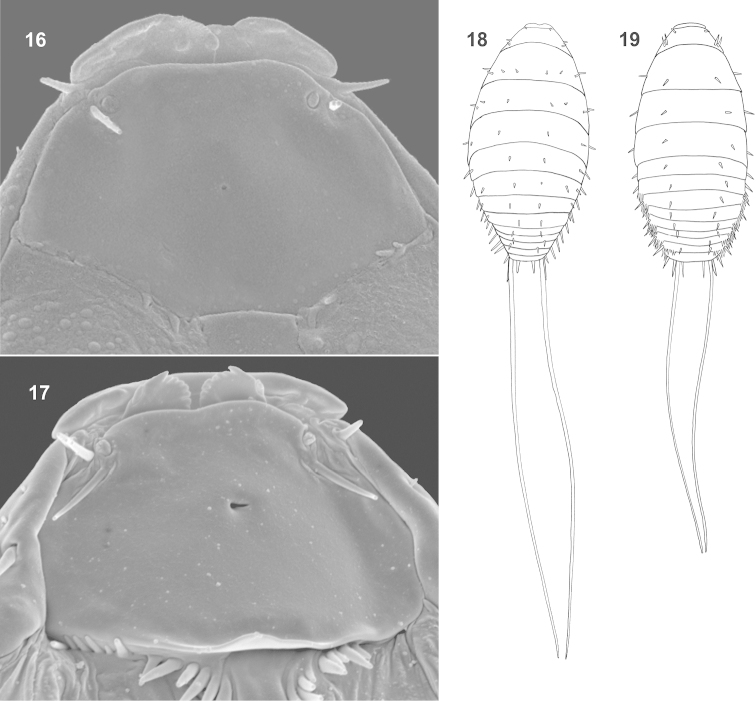
First instars, ventral view to head and dorsal view of total body. **16, 18** *Rozenia platicephala* sp. n. **17, 19** *Rozenia calliopsidis* sp. n.

#### Description.

**Female.** Canalis prolifer on abdominal segment I–IV. Tuba prolifera on segment IV positioned in posterior half of segment.

**Female puparium.** Cephalothorax slightly wider than long, approx. 0.7 mm long and approx. 0.8 mm wide between spiracular corners. Head wide, approx. 0.5 mm; mandible large, projecting from head contour, intermandibular distance 0.16–0.17 mm, mandibles approx. two mandibular diameters apart or less; labral apex between mandibles straight; oral ridge well developed; labral area very short; maxilla indistinct, but maxillary area with weak transverse elevation; brood opening wide, nearly from side to side, slightly sinuous, produced forward medially; head corners narrow, laterally turned posteriorly; posterolateral margin of head corner with weak apodeme; cephalic ridge well developed. Thorax without intersegmental ridges; pro-, meso- and metathorax largely fused ventrally as well as dorsally, segments seem to be subequal in length; thoracic stigma not developed; metathoracic ridge distinct, touching cintum and going up spiracle. Spiracular corners weakly prominent, obtuse; spiracula positioned anteriorly to spiracular corners, turned laterally; basal band distinct, arcuate, projecting forward, but anterior end not sharply delimited. Cephalothorax distinctly and uniformly light pigmented, only metathorax pale and translucent ventrally.

**First instar.** Total length (without caudal setae) 0.160–0.180 mm (n=6) on average; length of caudal setae up to 0.221 mm; ratio of body length and length of caudal seta 0.74–0.96. Ratio of body length and width approx. 2.2–2.3.

Head: Head dorsally with seven pairs of setae; ventrally strongly reduced, with setae on maxillae; mandibles and labrum overlapping outline of body; labrum not emarginated; labiomaxillary area occupying majority of ventral part of head, rounded, acute posteriorly.

**Figures 20–23.**
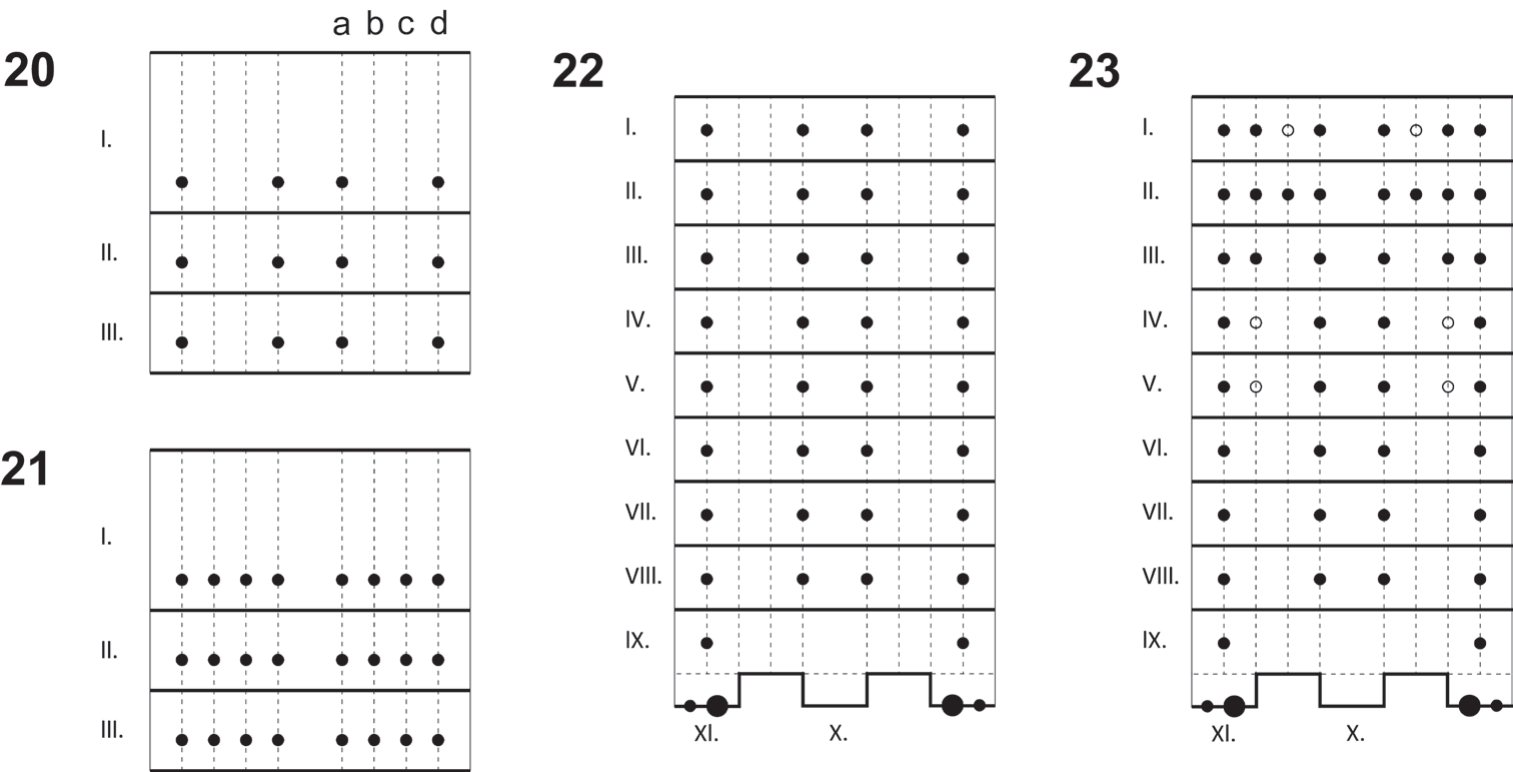
Diagram of first instar chaetotaxy. **20, 22** *Rozenia calliopsidis* sp. n., thoracic and abdominal tergites; **21, 23** *Rozenia platicephala* sp. n., thoracic and abdominal tergites; a-submedian row of setae, b-interstitial row of setae, c-supralateral row of setae, d-lateral row of setae; point-stable presence of seta; circle-seta with unstable presence; large point-caudal seta.

Thorax: Each segment of thorax bears two pairs of setae dorsally and laterally close to posterior margin, forming submedian and lateral rows of setae (Figure 20). Posterior margins of thoracic tergites smooth. Coxae broad and ovate; three coxal teeth at anterior part of each coxa, all variably divided into two to four tips; coxal bristle variably divided into four or five tips on fore leg and extensively bifid on mid and hind legs; single cuticular outgrowth positioned medially from coxal bristle; five cuticular outgrowths laterally from coxal teeth and one very short seta above cuticular outgrowths; one very short seta at the posterior part of coxa. Each trochanterofemur with spur bifid at tip, five to six cuticular outgrowths and one short seta anteriorly and posteriorly on femur. Each tibia with five tibial spurs and short projections at distal end of tibiae. Tarsi of fore and mid legs enlarged and elongated, tarsus of hind leg rod-like and elongated. Sternal figs broad and smooth on surface and with fringe of long spinulae at its posterior margin. Precoxal pleural membrane with transverse row of microtrichia on prothorax and with two processes laterally and medially on mesothorax and metathorax.

Abdomen: Abdomen with rows of setae dorsally and laterally similar to those present on thorax (Figure 22); submedian row of setae from abdominal tergite I to tergite VIII; lateral row of setae up to tergite IX. Abdominal segment X extremely shortened and fused to segment IX; segment XI split in two parts and restricted on ventral base of caudal setae; segment XI with one particularly long caudal seta and short lateral caudal seta. Posterior margin of abdominal tergites smooth except for a few spinulae (up to six) laterally, few setae present laterally as well as mesally from lateral row of setae; posterior margin of sternites with spinulae, segment IX with only two long spinulae, which extend body outline; no spinulae immersed.

#### Etymology.

Name derived from the generic name of the host bee.

#### Distribution.

Argentina and Chile.

### Rozenia peruana sp. n.

Taxon classificationAnimaliaStrepsipteraStylopidae

http://zoobank.org/84BFDD49-A710-48F1-A304-A4CA92138DCC

Figures 4 , 7 , 10

#### Material examined.

Holotype female puparium, in a separate microvial on same pin as host. Original label: “PERU: Lima dept. / Ricardo Palma, V-9-96 / J. G. Rozen, A. Ugarte”. 1 FP, host: *Acamptopoeum vagans* (Cockerell, 1926), ♀, JS Ascher det., AMNH coll. (code: AMNH_BEE 00026923).

#### Diferential diagnosis.

**Female puparium.** Cephalothorax of *Rozenia peruana* sp. n. strongly diverging posteriorly behind head (Figure 10). Among all the species of the genus *Rozenia peruana* sp. n. has the smallest mandibles that, as in other species, project from the head contour. A very specific character is the shape of prothorax in dorsal view. Prothorax produced forward on lateral sides to the head margin, thus posterior head margin is sinuous (Figure 10). Prothorax is dorsally pigmented as in *Rozenia platicephala* sp. n.

#### Description.

**Female.** Canalis prolifer on abdominal segment I–IV, with three large tuba prolifera on segments II-IV, tuba prolifera on segment I distinct, but very small and possibly not functional.

**Female puparium.** Cephalothorax slightly wider than long, approx. 0.7 mm long and approx. 0.9 mm wide between spiracular corners. Head wide, approx. 0.6 mm; mandible small, projecting from head contour, intermandibular distance 0.16 mm, mandibles nearly three mandibular diameters apart; labral apex between mandibles slightly arcuate; oral ridge well-developed; epipharinx weakly divided from very short labral area; maxilla indistinct; brood opening wide, nearly from side to side, arcuate; head corners narrow, directed posterolaterally; posterolateral margin of head corner with distinct apodeme; cephalic ridge weak. Thorax without intersegmental ridges; pro-, meso- and metathorax largely fused ventrally, segments seem to be subequal in length; prothorax dorsally slightly shorter than half length of fused meso- and metathorax, prothorax strongly produced forward laterally; metathorax as well as mesothorax laterally with remnant of stigmata; metathoracic ridge ill-defined, but distinct, touching cintum and going anterolaterally to spiracle. Spiracular corners prominent, triangular, well-developed; spiracle positioned anterior to spiracular corners, turned laterally; basal band arcuate, projecting forward, but ill-defined. All cephalothorax pale, head, prothorax dorsally, sides of thorax and spiracular corner light pigmented; rest of cephalothorax pale and translucent.

#### Etymology.

Name derived from the country, where the holotype was collected.

#### Distribution.

Peru.

### Rozenia platicephala sp. n.

Taxon classificationAnimaliaStrepsipteraStylopidae

http://zoobank.org/45297119-B4EB-407D-85A1-730D9A514903

Figures 2 , 5 , 8 , 11 , 13 , 15 , 16 , 18 , 21 , 23

#### Material examined.

Holotype female puparium, in a separate microvial on same pin as host. Original label: “CHILE: Cautin Prov. / Cunco, II-1998, / Perez de Arce”. 1 FP, host: *Acamptopoeum submetallicum* (Spinola, 1851), ♀, JS Ascher det., AMNH coll. (code: AMNH_BEE 00037984).

Paratypes: ARGentina: Córdoba prov.: Parral, Fundo Malcho, xi.1956, 1FP, host: *Acamptopoeum submetallicum* ♂, LE Peña leg., KUNHM coll. (SEMC1006814). Chile: same as holotype, >500 L1; Limarí prov., 19 km ENE Samo Alto, 10.xi.1992, 1FP, host: *Acamptopoeum submetallicum* ♀, JG Rozen, Sharkov and Snyder leg., AMNH coll. (AMNH_BEE 00037983); Cautin prov.: Cunco, ii.1998, 1 FP, host: *Acamptopoeum submetallicum* ♀, Perez de Arce leg., AMNH coll. (AMNH_BEE 00037985); Bio Bío prov.: Antuco, nr. Hydroeléctrica, 37°23'49"S, 71°27'21"W, 14.xii.2004, 1 FP, host: *Acamptopoeum submetallicum* ♂, JS Ascher leg., AMNH coll. (AMNH_BEE 00037986), ditto, 2 FPP, host: *Acamptopoeum submetallicum* ♀, JS Ascher leg., AMNH coll. (AMNH_BEE 00037391); Dichato, 20.xii.1953, 1 FP, >500 L1, host: *Acamptopoeum submetallicum* ♀, LE Peña leg., KUNHM coll. (SEMC1006914); Coquimbo prov.: Las Breas, 23.–24.x.1989, 1 FP, host: *Acamptopoeum submetallicum* ♀, JG Rozen leg., AMNH coll. (AMNH_BEE 00037393); Santiago prov.: El Manzano, Valle Rio, Maipo, 1000-1500 m, i.1984, 1 FP, host: *Acamptopoeum submetallicum* ♀, LE Peña leg., AMNH coll. (AMNH_BEE 00037394), El Manzano, Quebrada, 900-1500 m, 5.-6.ii.1983, 2 FPP, host: *Acamptopoeum submetallicum* ♀, LE Peña leg., AMNH coll. (AMNH_BEE 00037395); Valdivia prov.: Valdivia, 9.ii.1953, 1 FP, host: *Acamptopoeum submetallicum* ♀, collector not indicated, KUNHM coll. (SEMC1006957); Valparaíso prov.: Viňa del Mar, La Quinta Vergara, 18.xii.2004, 1 FP, host: *Acamptopoeum submetallicum* ♀, JS Ascher leg., AMNH coll. (AMNH_BEE 00037397). Other material examined: Coquimbo prov.: Las Breas, 23.–24.x.1989, 1 EMP, host: *Acamptopoeum submetallicum* ♂, JG Rozen leg., AMNH coll. (AMNH_BEE 00037392); Araucanía prov.: Malleco, Victoria, xii.1985, 1 EMP, host: *Acamptopoeum submetallicum* ♀, LE Peña leg., AMNH coll. (AMNH_BEE 00037396). All hosts identified by JS Ascher.

#### Diagnosis.

**Female puparium.** This species possess relatively and also absolutely the widest head among all species of the genus. Spiracular corners are sharply triangular and distinctly prominent, but not large. Prothorax is more pigmented dorsally than other parts of thorax, like in *Rozenia peruana* sp. n., but anterior and posterior margins are paralel, arcuate, producing forward on sides only slightly. Position of spiracula seems to be characteristic for this species. They are turned more dorsally than in other species, however this character is very variable and may be inconsistent. Pigmentation like in *Rozenia peruana* sp. n.

**First instar.** Shape of body is more rounded than in *Rozenia calliopsidis* sp. n., width of segments decreases from metathorax more strongly. Ratio of body length and width is on average 2.0. Ratio of body length and length of caudal setae is approx. 0.60–0.65. Caudal setae are relatively longest among all species.

Head dorsally with six pairs of setae compared to seven and usually longer in *Rozenia calliopsidis* sp. n.; labrum is narrow at the middle contrary to *Rozenia calliopsidis* sp. n.; labium is projecting more laterally than in *Rozenia calliopsidis* sp. n.

Each segment of thorax bears four pairs of setae dorsally and laterally, forming submedian, interstitial, supralateral and lateral rows of setae. Sternal figs are broad and smooth on surface, specific are also smooth posterior margins. Precoxal pleural membrane is smooth without any projections except of one or two cuticular outgrowths on prothoracic precoxal pleural membrane. Coxal teeth are always bifid in two tips in contrast to *Rozenia calliopsidis* sp. n. with as many as five tips; coxal bristle is always divided into two tips and bifurcation in middle leg and hind leg is not so extensive, there are no cuticular outgrowth by coxal bristle contrary to *Rozenia calliopsidis* sp. n., also there are not so many cuticular outgrowths on coxa and femur like in *Rozenia calliopsidis* sp. n.

All four pairs of rows of setae continues dorsally on abdomen, submedian row up to tergite XIII, interstitial row is on tergite II or in some specimens also on tergite I, supralateral row is variable and reach up to tergite III, IV or V, and lateral row up to tergite IX. Spinulae on posterial margins of abdominal tergites only beyond lateral row and not visible in dorsal view.

#### Description.

**Female.** Canalis prolifer on abdominal segment I–IV. Tuba prolifera on segment IV positioned in posterior half of segment.

**Female puparium.** Cephalothorax slightly wider than long, approx. 0.8 mm long and approx. 0.9–1.0 mm wide between spiracular corners. Head wide, approx. 0.7 mm; mandible projecting from head contour, intermandibular distance 0.17–0.21 mm, approx. two mandibular diameters apart, but variable among different individuals; labral apex between mandibles straight; oral ridge well developed; epipharinx weakly divided from labral area, short; maxilla not developed, but maxillary area with weak transverse elevation; brood opening wide, nearly from side to side, arcuate; head corners narrow, directed posterolaterally; posterolateral margin of head corner with distinct apodeme; cephalic ridge weak. Thorax without intersegmental ridges; pro-, meso- and metathorax largely fused ventrally, segments seem to be subequal in length, prothorax dorsally slightly shorter than half length of fused meso- and metathorax; meso- and metathorax laterally with remnants of stigma, mesothoracic spiraculum very small and hardly visible; metathoracic ridge ill-defined, but distinct, touching cintum and going up spiracle. Spiracular corners prominent, well developed; spiracula positioned anterior to spiracular corners, turned dorsally; basal band distinct but weak, arcuate, projecting forward. All cephalothorax pale, head and prothorax dorsally and head, prothorax and mesothorax light pigmented ventrally; spiracular area and basal band only slightly darker; rest of cephalothorax pale and translucent.

**First instar.** Total length approx. 0.154–0.175 mm (n=3) without caudal setae; length of caudal setae up to 0.289 mm (on an average 0.276 mm); ratio of body length and length of caudal setae approx. 0.60–0.65. Ratio of body length and width approx. 1.9–2.3.

Head: Head dorsally with six pairs of setae; ventrally strongly reduced; with setae on maxillae; distinctive mandibles and labrum overlapping outline of body; labrum emarginated; labiomaxillary area occupying majority of ventral part of head, rounded, posterior margin straight.

Thorax: Each segment of thorax bears four pairs of setae dorsally and laterally close to posterior margin forming submedian, interstitial, supralateral, and lateral rows of setae (Figure 21). Posterior margins of thoracic tergites smooth. Coxae broad and ovate; on each coxa three coxal teeth and one coxal bristle at anterior part of coxa, all bifid at tips; three or four cuticular outgrowths laterally from coxal teeth and one very short seta above cuticular outgrowths and one on posterior margin of coxa. Each trochanterofemur with femoral spur bifid at tip; two or three cuticular outgrowths and one short seta anteriorly and posteriorly on femur. Each tibia with five tibial spurs and little projections at distal end of tibiae. Tarsi of fore and middle legs enlarged and elongated, tarsi of hind legs rod-like and elongated. Sternal figs broad and smooth on surface and on posterior margins. Precoxal pleural membrane smooth without any projections except of one or two cuticular outgrowths on prothoracic precoxal pleural membrane.

Abdomen: Abdomen with rows of setae dorsally and laterally similar to those present on thorax; submedian row from abdominal tergite I to tergite VIII; interstitial row on tergite II or in some specimens also on tergite I; supralateral row variable up to tergite III, IV or V; lateral row up to tergite IX (Figure 23). Abdominal segment X extremely shortened and fused to segment IX; segment XI split in two parts and restricted only on ventral base of caudal setae; segment XI with particularly long caudal seta and short lateral caudal seta. Posterior margin of abdominal tergites smooth except for lateral part with a few spinulae (up to three) more laterally than lateral row of setae; posterior margin of sternites with spinulae, segment IX with only two longer spinulae, which extend body outline; no spinulae immersed.

#### Etymology.

Name of this species refers to characteristic flat head and general flat appearance of all *Rozenia* gen. n. species, when found between tergites of host bees.

#### Distribution.

Argentina and Chile.

#### Published hosts assigned to *Rozenia platicephala* sp. n.

*Acamptopoeum submetallicum*: Schwarz (1931: 78-79), record from Chile (as *Liopoeum submetallicum* (Spinola)), also reported by Hofeneder and Fulmek (1943: 35), but with no original data.

#### Note

To *Rozenia platicephala* sp. n. could be assigned findings of Strepsiptera in the host bee *Acamptopoeum argentinum* (Friese, 1906): Hofeneder and Fulmek (1943: 42), record from Argentina (as *Perdita argentina* Friese), repeated by Hofeneder (1949: 122) and later by Hofeneder (1952: 489) (as Calliopsis (Parafriesea) argentina (Friese)). The record is impossible to verify as reliable pending a review of the material. The information about material deposition is not known to us.

### Key to species of the genus *Rozenia* gen. n.

Female puparia and females

**Table:** 

1a	More than four abdominal segments developed, with tuba prolifera III (if developed) positioned in the middle part of abdominal segment IV; combination of characters different	other Strepsiptera
1b	Only four abdominal segments developed, with tuba prolifera III positioned on the posterior half of abdominal segment IV (Figures 1–2); brood opening wide, almost from side to side, about four times wider than intermandibular distance, or more; head wider than distal part of prothorax, this character cause that side of cephalothorax is not continuously diverging posteriorly; mandibles extending from the head contour in ventral view; intersegmental ridges not developed (Figures 3–11)	2, *Rozenia* gen. n.
2a	Spiracular corners weakly prominent, obtuse, not triangular; brood opening turned backwards laterally, very close to posterior margin of mandible and continued as cephalic ridge (Figures 3, 6); cephalothorax pigmented in all parts (Figure 3); host bee *Calliopsis* spp.	*Rozenia calliopsidis* sp. n.
2b	Spiracular corners prominent, triangular; brood opening fluently transforms into cephalic ridge and forms an arcuate line (Figures 4, 5, 7, 8); posterior half of cephalothorax nearly transparent (Figures 4, 5); host bee *Acamptopoeum* spp.	3
3a	Prothorax dorsally produced forward on lateral sides to the head margin, thus posterior head margin is sinuous (Figure 10); mandibles very small (Figures 4, 7)	*Rozenia peruana* sp. n.
3b	Anterior and posterior margins of prothorax dorsally parallel, thus posterior head margin arcuate (Figure 11); mandibles of normal size (Figures 5, 8)	*Rozenia platicephala* sp. n.

**First instars**

**Table:** 

1a	Submedian row of setae absent on abdomen; caudal setae shorter or as long as body; posterior margin of labium emarginated; ventral sublateral bristle on sternite IX; posterior margin of thoracic tergites with spinulae	other Strepsiptera
1b	Submedian row of setae present on abdomen; caudal setae longer then body; posterior margin of labium continuous; ventral sublateral bristle absent; posterior margin of thoracic tergites smooth	2, *Rozenia* gen. n.
2a	Sternal figs at posterior margin with spinulae; interstitial and supralateral row of setae on dorsum absent; coxal tooth with two to four tips at apex; coxal bristle in fore leg with multiple tips at apex; coxal bristles in mid and hind leg extensively bifid; numerous cuticular outgrowths on precoxal pleural membrane and coxae; caudal setae slightly longer then body	*Rozenia calliopsidis* sp. n.
2b	Sternal figs smooth on posterior margin; interstitial and supralateral row of setae on dorsum; coxal tooth bifid at apex; coxal bristles bifid on each leg; few cuticular outgrowths on precoxal pleural membrane and coxae; caudal setae distinctly longer then body	*Rozenia platicephala* sp. n.

## Discussion

Among all, the newly described genus, *Rozenia* gen. n., is morphologically unusual in having extremely long caudal setae in first instars. No other Strepsiptera species possess such long caudal setae (Pohl 2000). These setae are always longer than the body in *Rozenia* gen. n., and almost two times longer than the body in *Rozenia platicephala* sp. n. (Figures 18–19). This species is also exceptional in having four rows of dorsal thoracic setae, one row more than in the most basal Strepsiptera family Mengenillidae (Pohl 2000). Until now, the chaetotaxy of first instars seemed to be reductive in the evolution of Strepsiptera, because basal lineages possess more abundant setae on dorsal part of the thorax and abdomen than derived lineages. It is, however, clear that *Rozenia* gen. n. is not related to the Mengenillidae, but belongs to the family Stylopidae, which means that at least one row of setae are newly developed in *Rozenia* gen. n. We call the new row of setae the “interstitial row”, because at most three rows of setae were known in all other Strepsiptera untill now. This interstitial row continues to abdominal segments I and II in *Rozenia platicephala* sp. n. The second species of *Rozenia* gen. n. with known first instars, *Rozenia calliopsidis* sp. n., has a more standard chaetotaxy, but a submedian row of setae is present on the thorax, as well as on abdominal segments I-VIII, which is a synapomorphy of the genus *Rozenia* gen. n.

*Rozenia* gen. n. is a genus distinctive from other Strepsiptera genera in numerous characters mentioned in generic diagnosis. According to the host family and a few shared characters, it seems to be most closely related to the genus *Crawfordia*. In both genera, a single median tuba prolifera on canalis prolifer is present on segments II-IV of the abdominal part of female puparia. In first instars, spinulae are not immersed in any part of the body; two pairs of setae or more are present on each thoracic and abdominal segment dorsally; the sternal figs are completely smooth, no setae are developed; coxal teeth, coxal bristles and femoral spurs are bifid or with multiple tips in both genera. Some of these characters are developed in some other Strepsiptera species, but never in the family Stylopidae (Pohl 2000).

## Supplementary Material

XML Treatment for Rozenia

XML Treatment for Rozenia calliopsidis

XML Treatment for Rozenia peruana

XML Treatment for Rozenia platicephala
